# The influence of transpiration on foliar accumulation of salt and nutrients under salinity in poplar (*Populus* × *canescens*)

**DOI:** 10.1371/journal.pone.0253228

**Published:** 2021-06-24

**Authors:** Shayla Sharmin, Ulrike Lipka, Andrea Polle, Christian Eckert

**Affiliations:** Forest Botany and Tree Physiology, University of Göttingen, Göttingen, Germany; Beijing Forestry University, CHINA

## Abstract

Increasing salinity is one of the major drawbacks for plant growth. Besides the ion itself being toxic to plant cells, it greatly interferes with the supply of other macronutrients like potassium, calcium and magnesium. However, little is known about how sodium affects the translocation of these nutrients from the root to the shoot. The major driving force of this translocation process is thought to be the water flow through the xylem driven by transpiration. To dissect the effects of transpiration from those of salinity we compared salt stressed, ABA treated and combined salt- and ABA treated poplars with untreated controls. Salinity reduced the root content of major nutrients like K^+^, Ca^2+^ and Mg^2+^. Less Ca^2+^ and Mg^2+^ in the roots resulted in reduced leaf Ca^2+^ and leaf Mg^2+^ levels due to reduced stomatal conductance and reduced transpiration. Interestingly, leaf K^+^ levels were positively affected in leaves under salt stress although there was less K^+^ in the roots under salt. In response to ABA, transpiration was also decreased and Mg^2+^ and Ca^2+^ levels decreased comparably to the salt stress treatment, while K^+^ levels were not affected. Thus, our results suggest that loading and retention of leaf K^+^ is enhanced under salt stress compared to merely transpiration driven cation supply.

## Introduction

Soil salinity is one of the most severe abiotic stress that limits the distribution and productivity of crops worldwide. Salinization of arable soils can have natural causes but is mostly the consequence of unsuitable cultivation practices [1, 2]. Soils are generally classified as saline when the electrical conductivity of the saturated soil extract is 4 dS m^-1^ or more [3], equivalent to approximately 40 mM NaCl [4]. The presence of soluble salts at higher concentrations in the soil reduces water availability to roots and causes ion toxicity and nutrient deficiency in plants [1, 5, 6].

Plants acquire nutrients from the environment surrounding their root system. Under salinity, Na^+^ and Cl^-^ can disrupt nutrient uptake of glycophytes through competitive interactions or by affecting the membrane selectivity for ions [7]. The presence of NaCl under saline conditions results in nutritional imbalances inside the plant evident as high ratios of Na^+^/Ca^2+^, Na^+^/K^+^ and Na^+^/Mg^2+^ [8–10]. After uptake by the roots, the delivery of ions from roots to leaves occurs through the vascular system of the xylem with the transpiration stream as the transport vehicle [11]. Since the movement of ions from root to shoot is influenced by the transpiration-driven water flow [12], shoot ion uptake is affected by both the ion concentration and the rate of transpiration.

Among woody plants, poplars (*Populus* spp.) have often been used to investigate the responses to salt stress [2]. These studies suggested a role of abscisic acid (ABA) in restricting salt uptake [13, 14]. It is well known that ABA is a central regulator of plant adaptation to osmotic stress [15]. ABA regulates stomatal opening [16, 17]. The levels of ABA in poplars increase in response to salinity [18–21]. Overall, higher levels of ABA in salt-tolerant compared to salt-sensitive hybrid [(*P*. *euphratica* versus *P*. *talassica* Kom × (*P*. *euphratica* + *Salix alba* L.)] under stress conditions suggested that ABA-induced stomatal closure may reduce root-to-shoot xylem water flow and consequently limit the total amount of salt ions transported to leaves [13]. However, enhanced salt accumulation in roots and elevated xylem loading may counteract the anticipated ameliorating effect of a reduced transpirational pull on foliar salt accumulation. It is thus still unclear, whether ABA contributes to decreasing tissue salt enrichment.

In addition to salt accumulation, exposure to enhanced NaCl causes alteration in tissue concentrations of cationic nutrients such as K^+^, Ca^2+^ and Mg^2+^ [20]. For example, in a poplar hybrid NaCl treatment caused reductions in Mg^2+^ and Ca^2+^ levels in roots and leaves, while K^+^ level was unaffected [13]. In some other poplar genotypes, e.g., *P*. *tomentosa* and *P*. × *canescens*, salinity caused reduction in K^+^ uptake in roots but no reduction in leaves [19, 21]. Salinity induced reduction in the tissue concentrations of K^+^, Ca^2+^ and Mg^2+^ nutrients was also reported in other salt-sensitive woody plants e. g. in citrus rootstocks, avocado rootstocks, cherry etc. [22–25]. These examples show that it is not known if the reduction of nutrients in tissues under salinity is predominantly due to less uptake by root or if the reduced transport via the transpiration stream plays role as well.

This study aimed to investigate the effect of reduced transpiration on nutrient accumulation in *P*. × *canescens* under salt stress. We hypothesize that reduced transpiration decreases accumulation of Na^+^ and contributes to a favorable ion balance in leaves, thereby, protecting poplars against salinity stress. In addition, we hypothesize that accumulation of Na^+^ and other cations is independent from stomatal opening and therefore, enhanced NaCl content in soils imposes Na^+^ accumulation independent of transpiration. Gas exchange, growth, ion concentrations in leaves and root were measured in *P*. × *canescens* and the resultant effect on the growth of the plants was analyzed as well. Our results suggest that interplay between foliar ion accumulation and transpiration is moderate.

## Materials and methods

### Plant material

Plantlets of *P*. × *canescens* (clone INRA717 1-B4) were multiplied by *in-vitro* micropropagation as described by Leplé and colleagues [26]. Approximately, 1 to 2 cm long stem cuttings having at least one leaf were placed up-right into glass jars containing half strength Murashige & Skoog (MS) medium [27] under sterile conditions and incubated in a culture room [16 h light / 8 h dark, 150 μmol PAR m^-2^ s^-1^ (Osram L 18W/840 cool white, Osram, Munich, Germany), 23 to 25°C, 40 to 60% relative air humidity] for 5 weeks as described by Müller and colleagues [28]. Afterward, rooted plants were moved into a greenhouse (Department of Forest Botany and Tree Physiology, University of Göttingen, Göttingen, Germany), acclimated to ambient conditions, and raised in aerated hydroponic culture with Long-Ashton (LA) nutrient solution [28]. The plants were grown with additional light (150 μmol m^-2^ s^-1^ PAR) (Lamp: 3071/400 HI-I, Adolf Schuch GmbH, Worms, Germany) to maintain a 16 h photoperiod and at air temperatures from 21 to 24°C and relative air humidity from 70 to 80%. The nutrient solution was exchanged weekly. After a growth phase of 5 weeks, when the plants had mean height of 29.82 ± 4.24 cm, the experimental treatments were started in September 2017. The experiment was conducted with a total of 94 plants.

### Salt and ABA treatment

The total duration of the treatment period was six weeks (S1 Fig). Before applying saline stress, the plants were divided into three groups: control, low salt and ABA [(±) ABA, Duchefa Biochemie B.V, Haarlem, Netherlands]. The control group was supplied with LA nutrient solution as before. The low salt (Ls) group was exposed to 25 mM NaCl in the nutrient solution. The ABA group was exposed to 10 μM ABA for 1 week and then to 50 μM ABA for 2 weeks in the nutrient solution (S1 Fig).

After three weeks, groups were divided into the following experimental groups (S1 Fig): The control group was split into two groups, of which one was kept under control conditions (control) and the second was stressed with 100 mM NaCl (Hs group). The low salt group was divided into two groups, of which one was kept with 25 mM NaCl [continuous low salt (cLs) group] and the second was exposed to 100 mM NaCl (Ls+Hs group). The ABA treated group was split in four treatments, among which one group was returned to control conditions in LA nutrient solution [discontinuous ABA (dABA) group]; in the second group, 50 μM ABA treatment was continued [continuous ABA (cABA) group]; the third group was exposed to 100 mM NaCl only [discontinuous ABA plus high salt (dABA+Hs) group] and the fourth group was exposed to 100 mM NaCl together with 50 μM ABA [continuous ABA plus high salt (cABA+Hs) group]. This resulted in a total of eight different treatments (S1 Fig). The nutrient solutions with different salt or ABA amendments as well as the control solutions were exchanged weekly. The plants were randomized regularly. After three weeks of stress phase, all plants were harvested (n = 9 to 10 per treatment).

### Plant growth measurements

Plant height was recorded twice a week and stem diameter was recorded once a week. The shoot height was measured from the growing tip to the base of the stem. The stem diameter was measured by a digital caliper at a marked position approximately 2 cm above from the base of the plant. Relative height increment and relative diameter increment over the stress phase i.e., last three weeks of the whole-treatment period were calculated by the following formula:

$$Relative\;height\;increment=\frac{H_{\mathrm{end}}‐H_{\mathrm{start}}}{H_{\mathrm{start}}}$$

$$Relative\;diameter\;increment=\frac{D_{\mathrm{end}}‐D_{\mathrm{start}}}{D_{\mathrm{start}}}$$

Where *H*_*start*_ and *H*_*end*_ are the shoot heights, and *D*_*start*_ and *D*_*end*_ are the shoot diameters at the time of start and end of stress phase, respectively.

Shed leaves per individual plant were collected during the six-week-long experimental period and dried at 60°C for 7 days to determine total leaf loss.

### Gas exchange measurement

Net photosynthesis, transpiration, stomatal conductance, sub-stomatal CO_2_ concentration and atmospheric CO_2_ concentration of mature leaves (using the 8^th^ -10^th^ leaf from the apex) were measured once a week between 9:00 h and 14:00 h with an LCpro+ portable photosynthesis system (ADC BioScientific Ltd., Hoddesdon, UK). The measurements were carried out with constant irradiation of 870 μmol PAR m^-2^ s^-1^ and a temperature of 23.1 ± 0.6 ⁰C and at 419.7 ± 11.2 μmol mol^-1^ ambient atmospheric CO_2_ concentration.

### Harvest

At the end of the experiment, destructive harvest was done. Leaves, stem and root of each plant were weight separately. The dry mass was determined after drying aliquots from each tissue for 7 days at 60°C. The dry mass of the whole tissue was calculated as:

$$Total\;tissue\;dry\;mass\;(g)=\frac{\mathrm{total}\;\mathrm{fresh}\;\mathrm{mass}\;\mathrm{of}\;\mathrm{the}\;\mathrm{tissue}\;(g)\times \mathrm{dry}\;\mathrm{mass}\;\mathrm{of}\;\mathrm{the}\;\mathrm{aliquot}\;(g)}{\mathrm{fresh}\;\mathrm{mass}\;\mathrm{of}\;\mathrm{the}\;\mathrm{aliquot}\;(g)}$$

For leaf area measurement, three leaves from the top, middle and bottom part of the shoot were collected, weighed, and scanned. The area of each leaf was measured from scanned pictures using ImageJ software. Leaf size and whole-plant leaf area were calculated using the following equations:

$$Leaf\;size\;(cm^{2}\;leaf^{‐1})=\frac{\mathrm{leaf}\;\mathrm{area}\;\mathrm{of}\;\mathrm{sample}\;\mathrm{leaves}\;(m^{2})}{\mathrm{number}\;\mathrm{of}\;\mathrm{sample}\;\mathrm{leaves}}\times 10000$$

$$Whole‐plant\;leaf\;area\;(m^{2}\;plant^{‐1})=\frac{\mathrm{leaf}\;\mathrm{area}\;\mathrm{of}\;\mathrm{sample}\;\mathrm{leaves}\;(m^{2})\times \mathrm{fresh}\;\mathrm{mass}\;\mathrm{of}\;\mathrm{all}\;\mathrm{leaves}\;\mathrm{of}\;\mathrm{the}\;\mathrm{plant}\;(g)}{\mathrm{fresh}\;\mathrm{mass}\;\mathrm{of}\;\mathrm{the}\;\mathrm{sample}\;\mathrm{leaves}\;(g)}$$

### Analysis of elements

Different elements were measured in the representative aliquots of leaves and fine roots of a plant (5 or 10 plants per treatment). Dried aliquots of leaf and root tissues were milled (Retsch, Haan, Germany) into fine powder before digestion. Approximately 40 to 50 mg of ground sample was digested with 2 ml of 65% HNO_3_ in a microwave digestion system (ETHOS.start, MLS GmbH, Leutkirch, Germany). The microwave program used for the digestion of sample was as follows: 2.5 min at 90°C (power 1000 W), 5 min at 150°C (power 1000 W), 2.5 min at 210°C (power 1600 W) and 20 min at 210°C (1600 W). The resulting solutions were cooled and filled up to 25 ml volume with de-ionized water. The final volumes were filtered by filter paper (MN 640 w, 90 mm, Macherey-Nagel GmbH & Co. KG, Düren, Germany) and elements (Na, K, Ca, Mg, S, Mn, Fe and P) were measured in the filtered extracts by inductively coupled plasma-optical emission spectrometry (ICP-OES) (iCAP 7000 series ICP-OES, Thermo Fisher Scientific, Dreieich, Germany). The element concentrations (mg g^-1^ dry mass) were calculated using calibration standards (Single-element standards, Bernd Kraft GmbH, Duisburg, Germany) and the sample weight used for extraction.

To determine a hypothetical concentration of ions in the cellular fluid (assuming equal distribution of the ions throughout the cell), measured elements were expressed on the basis of water content of the tissue (mM). The water content and ion concentration were calculated as follows:

$$Water\;content\;(L\;g^{‐1}\;dry\;mass)=\frac{\mathrm{total}\;\mathrm{fresh}\;\mathrm{mass}\;\mathrm{of}\;\mathrm{the}\;\mathrm{tissue}‐\mathrm{total}\;\mathrm{dry}\;\mathrm{mass}\;\mathrm{of}\;\mathrm{the}\;\mathrm{tissue}}{\mathrm{total}\;\mathrm{dry}\;\mathrm{mass}\;\mathrm{of}\;\mathrm{the}\;\mathrm{tissue}}$$

$$Concentration\;of\;ion\;(mM)=\frac{\mathrm{concentration}\;\mathrm{of}\;\mathrm{element}\;(\mathrm{mg}\;g^{‐1}\;\mathrm{dry}\;\mathrm{mass})\;\mathrm{in}\;\mathrm{the}\;\mathrm{tissue}}{\mathrm{atomic}\;\mathrm{mass}\;\mathrm{of}\;\mathrm{that}\;\mathrm{element}\;\times \;\mathrm{water}\;\mathrm{content}\;\mathrm{of}\;\mathrm{the}\;\mathrm{tissue}(L\;g^{‐1}\;\mathrm{dry}\;\mathrm{mass})}$$

The relative changes in the concentration of major cations (K^+^, Ca^2+^ and Mg^2+^) in response to different treatments were calculated by comparing with controls as follows:

$$Change\;in\;the\;concentration\;of\;ion\;(\%)=\frac{\mathrm{concentration}\;\mathrm{of}\;\mathrm{ion}\;(\mathrm{mM})‐\mathrm{mean}\;\mathrm{concentration}\;\mathrm{in}\;\mathrm{control}\;(\mathrm{mM})}{\mathrm{mean}\;\mathrm{concentration}\;\mathrm{in}\;\mathrm{control}\;(\mathrm{mM})}\times 100$$

### Scanning electron microscope (SEM) and energy-dispersive x-ray microanalysis (EDXA)

Electron microscopy and X-ray microanalysis were done in root tips from selected treatments (3 or 4 plants per treatment). Two to three fresh root tips (approx. 1 cm long) per plant were harvested, wrapped with aluminum foil paper and enclosed in mesh wire bags (Haver and Boecker, Oelde, Germany). A freezing mixture of propane: isopentane (2:1) was prepared in a small container at the temperature of liquid nitrogen [29]. The wire bags containing root samples were immediately dipped into the freezing mixture for 2 to 3 min. Afterwards, the bags were transferred into liquid nitrogen for further storage.

To prepare a frozen root tip for electron microscopy, a small portion of a root tip (1 mm above from root apex) was cut off and removed with a thin razor blade, and the cut surface was fixed firmly using freeze adhesive (Tissue freezing medium, Leica Biosystems, Nussloch, Germany) on the holder of the electron microscope at the temperature of liquid nitrogen. The holder and sample were then clamped in the cooling stage of the microscope (-25°C). Samples were analyzed by a scanning electron microscope (Phenom ProX, Phenom-World B.V., Eindhoven, Netherlands) equipped with an energy dispersive spectrometer (EDS) and element identification (EID) software package. The acceleration voltage of 15 kV and magnifications of 300X, 350X and 2500X were used with an acquisition time of 55 seconds. Element distribution was analyzed across the root cell layers radially from the center of the vascular cylinder to the rhizodermis. For this purpose, line scans with 512 pixels resolution were analyzed. To obtain representative data, at least four line-scan analyses at four separate positions per sample were recorded. Relative concentration (percentage of weight) of different elements (Na, K, Ca, Mg, P, Mn, S and Cl) obtained from line scan analyses were separated based on the distribution within cortex and vascular cells for further comparison.

### Statistical analysis

Statistical analyses were performed with the statistical software R (version 3.5.2). One-way and two-way analysis of variance (ANOVA) was applied followed by Fisher’s test. Normal distribution of data was tested by plotting residuals and log transformation or square root transformation was used if data were not normally distributed. Data represent means ± standard error (SE). If not indicated otherwise, n = 5 biological replicates were investigated. Means were considered to be significantly different when p ≤ 0.05.

## Results

### Gas exchange of plants decreases strongly in response to high salt and moderately in response to ABA

**Fig 1:**
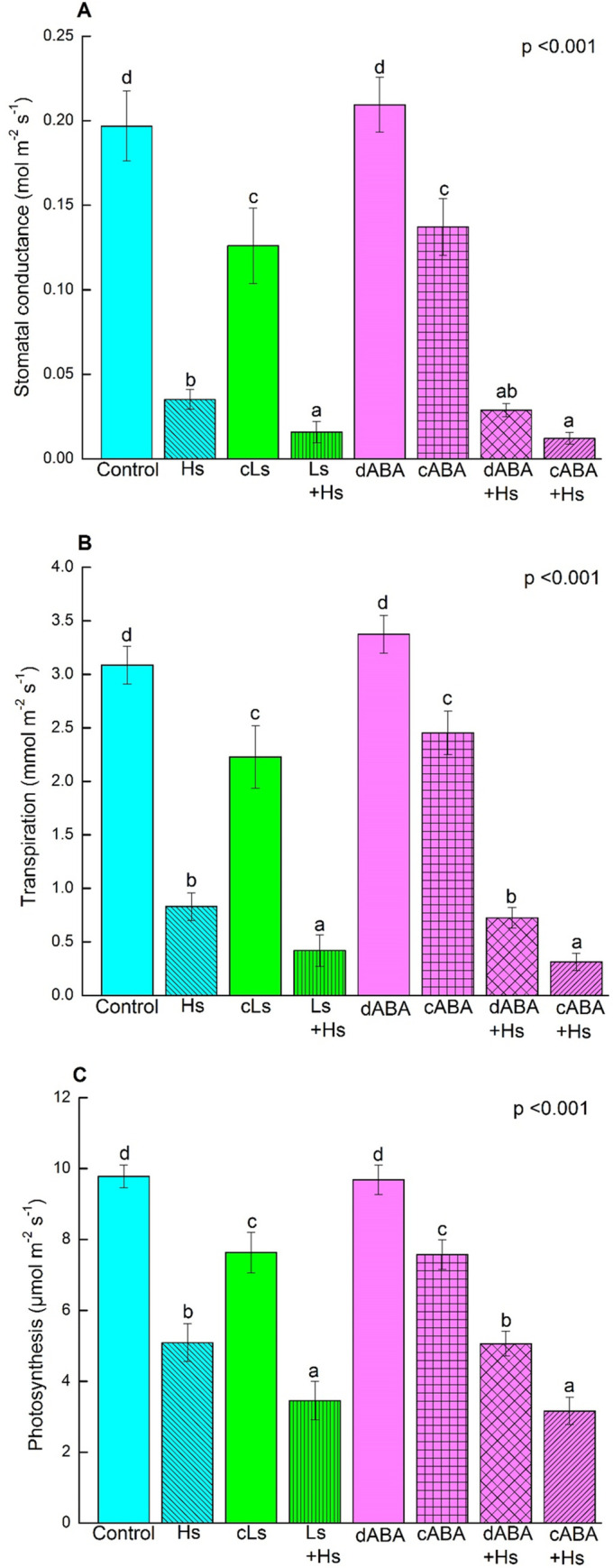
Stomatal conductance (A), transpiration (B) and photosynthetic rate (C) in the leaf of *P*. × *canescens* plants in response to salinity and ABA treatment. Bars indicate means ± SE. Data were obtained by analyzing all measurements from five independent plants per treatment (once a week over three weeks of stress period). One-way ANOVA was conducted. Normal distribution of data was tested by plotting residuals. Different letters obtained from Fisher’s test indicate significant differences among treatments at p <0.05. ***Control*** = constantly grown with nutrient solution only; ***Hs =* high salt** i.e. exposed to 100 mM NaCl for three weeks of stress phase; ***cLs*** = **continuous low salt** i.e. applied with 25 mM NaCl constantly for six weeks of whole-treatment phase; ***Ls + Hs*** = **low salt plus high salt** i.e. applied with 25 mM NaCl for three weeks of pretreatment phase and then replaced with 100 mM NaCl for next three weeks of stress phase; ***dABA*** = **discontinuous ABA** i.e. treated with 50 μM ABA for three weeks of pretreatment phase and then replaced with only nutrient solution for next three weeks of stress phase; ***cABA*** = **continuous ABA** i.e. treated with 10 μM ABA in first week and then with 50 μM ABA constantly in the next five weeks of whole-treatment period; ***dABA + Hs*** = **discontinuous ABA plus high salt** i.e. treated with 50 μM ABA for three weeks of pretreatment phase and then replaced with 100 mM NaCl for next three weeks of stress phase; ***cABA + Hs*** = **continuous ABA plus high salt** i.e. 50 μM ABA was applied for three weeks of pretreatment phase and then 50 μM ABA plus 100 mM NaCl was applied for three weeks of stress phase.

Poplars exposed to high salt (Hs, 100 mM NaCl) showed an about 5-fold decline in stomatal conductance and transpiration (Fig 1A and 1B) and an about 2-fold decline in net CO_2_ assimilation (Fig 1C) compared to control plants. Low salt treatment (cLs, 25 mM NaCl) resulted in less pronounced decreases in gas exchange compared to high salt (Fig 1A–1C). When the low salt pretreated plants were transferred to high salt conditions (Ls+Hs), the negative impact of salt was even stronger than in absence of low salt pretreatment (Hs, Fig 1A–1C).

Exposure of poplars to 50 μM ABA (cABA) had a negative influence on gas exchange similar to that observed in response to low salt stress (cLs, Fig 1A–1C). The ABA effect was fully reversible when the poplars were transferred after an ABA pretreatment phase to control nutrient solution (dABA, Fig 1A–1C). When poplars grown in the presence of ABA were exposed to high salt (cABA+Hs), the decline in stomatal conductance, transpiration and net photosynthesis was similar to that of plants exposed to high salt after low salt pretreatment (Ls+Hs, Fig 1A–1C). These treatments had the strongest negative effects on gas exchange. Plants, which were exposed to high salt with the discontinuation of ABA application (dABA+Hs) showed a decline in gas exchange similar to that of plants exposed only to high salt (Hs, Fig 1A–1C). Overall, exposure to high salt stress resulted in a stronger decline in gas exchange than that to either low salt stress or ABA treatment.

### Shoot growth reduces significantly after salt exposure but leaf area declines in all stress treatments

**Fig 2:**
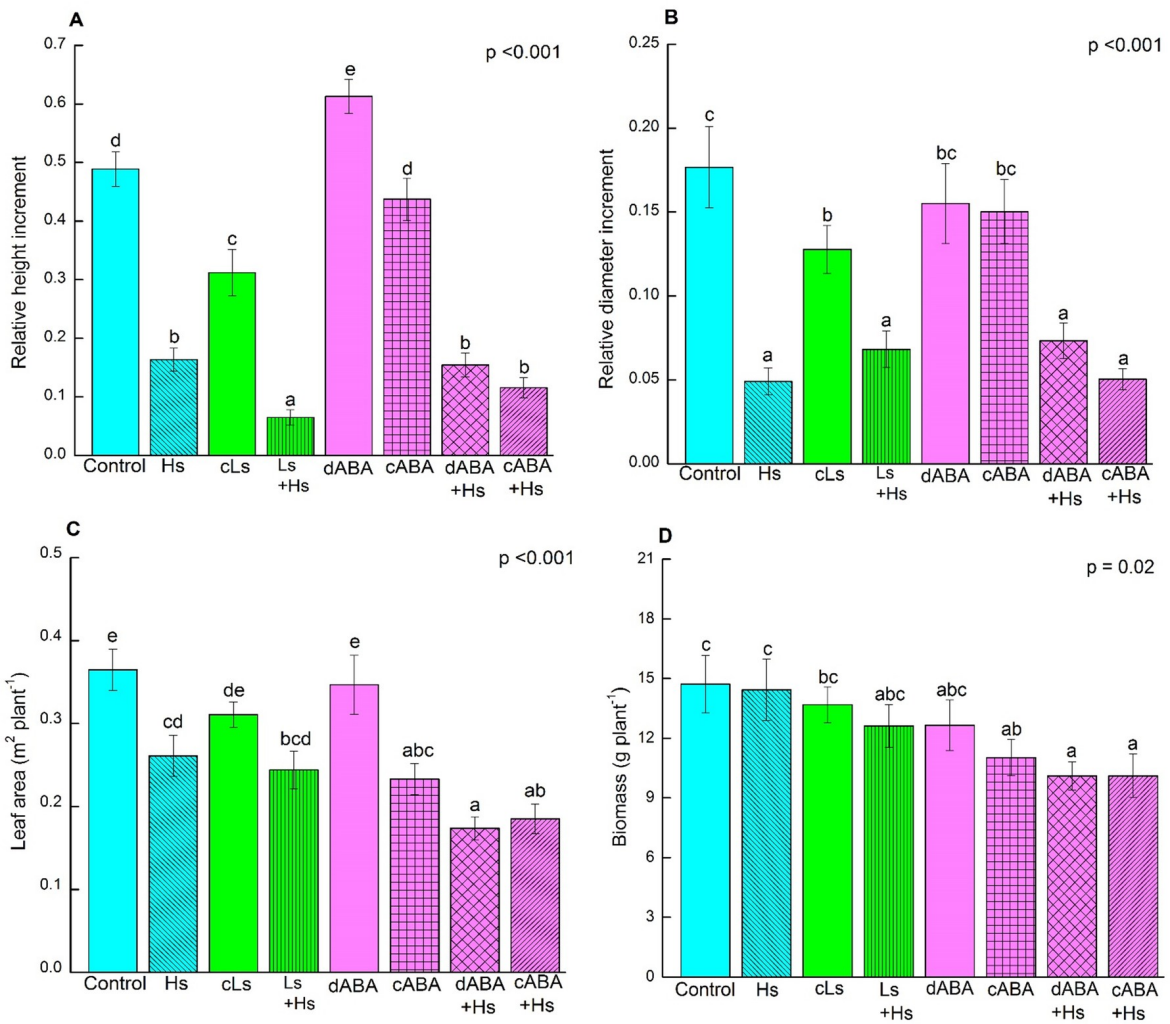
Relative shoot height increment (A), relative shoot diameter increment (B), leaf area (C) and biomass accumulation (D) of *P*. × *canescens* plants under salinity and ABA. Bars indicate means ± SE (n = 9 or 10; in case of leaf area, n = 7 or 8). One-way ANOVA was conducted. Normal distribution of data was tested by plotting residuals and square root transformation (in case of relative height increment) or log transformation (in case of biomass) was used to meet the criteria. Different letters obtained from Fisher’s test indicate significant differences among treatments at p <0.05.

The influence of salinity and ABA on the growth of the plants was investigated by examining relative shoot height increment, stem diameter increment, leaf area and biomass (Fig 2). Relative shoot height and stem diameter increments were significantly lower under high salt treatments, irrespective of with or without ABA (Fig 2A and 2B). Low salt exposure reduced height growth and diameter increment significantly (Fig 2A and 2B). ABA treatments caused no significant alterations in either height or diameter increment (Fig 2A and 2B). Whole-plant leaf area decreased significantly in response to salt stress as well as to ABA application (Fig 2C). The size of individual leaves was also reduced significantly in all stress treatments (S1 Table). Whole-plant biomass (sum of root, stem and leaves) showed a significant reduction in biomass for all ABA and high salt treated plants (Fig 2D). Loss of biomass due to leaf shedding was found in non-stressed and stressed plants and there was no significant difference among the treatments (S1 Table). However, plants showed tendency towards higher leaf loss in response to high salt and ABA exposure compared to control conditions (S1 Table). The root to shoot ratio was increased marginally in the presence of high salt and ABA, though difference was not significant (S1 Table).

### Basic cation concentrations are altered in roots and leaves in response to salinity and ABA

**Fig 3:**
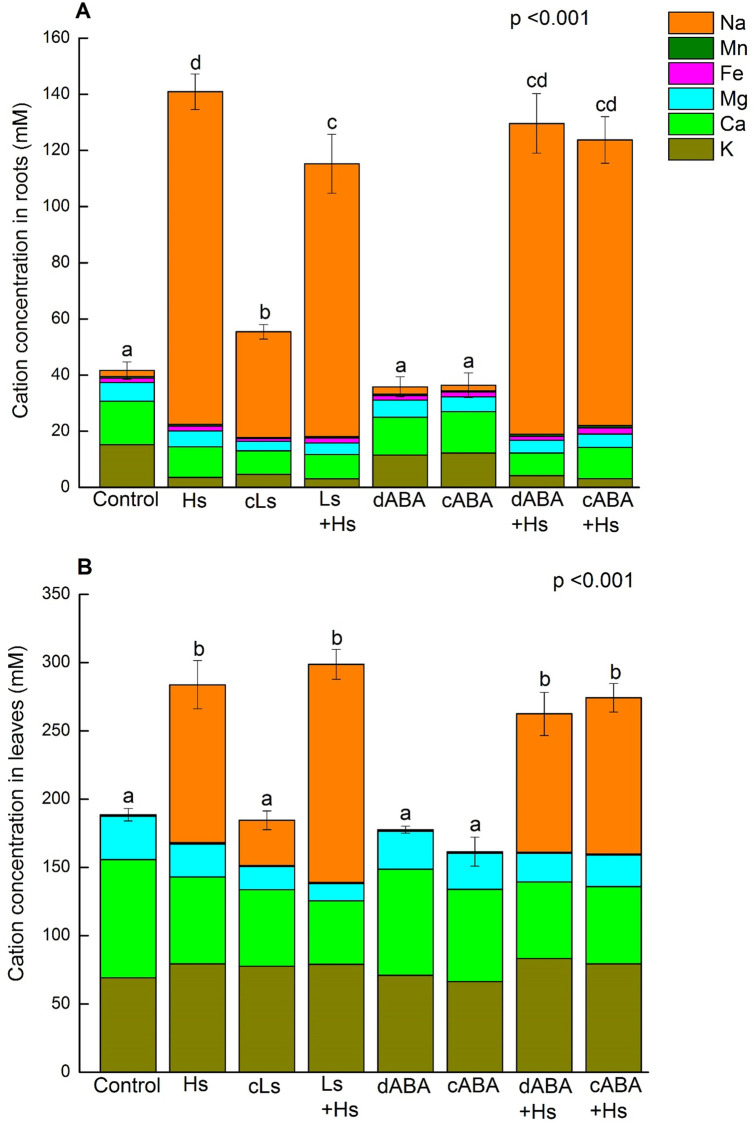
Concentration of cations in the root (A) and leaf tissues (B) of *P*. × *canescens* plants in response to salinity and ABA. Cations (mM) were calculated as sum of K^+^, Ca^2+^, Mg^2+^, Fe^2+^, Mn^2+^ and Na^+^. Bar represents means ± SE (n = 5 or 10). Average content of specific ion is presented by specific color on the bar. One-way ANOVA was conducted. Normal distribution of data was tested by plotting residuals and log transformation was used in case of cation content in root. Different letters obtained from Fisher’s test indicate significant differences among treatments at p <0.05.

To obtain information how salt stress or ABA treatments affected the ion balance, we estimated the total cation concentrations on the basis of the water content of root or leaf tissues (Fig 3). High salt exposure caused approximately 3- to 3.5-fold increases in root cation concentrations compared to controls (Fig 3A), whereas the increase in leaves was approximately 1.5-fold (Fig 3B). The increase was caused by substantial accumulation of Na^+^ and partly counterbalanced by decreases in other cations (Fig 3A and 3B). The contributions of the micronutrient Mn and Fe to these alterations were negligible (Fig 3A and 3B). Details for all measured elements and their ratios are available in S2 and S3 Tables for root tissue and S4 and S5 Tables for leaf tissue.

**Fig 4:**
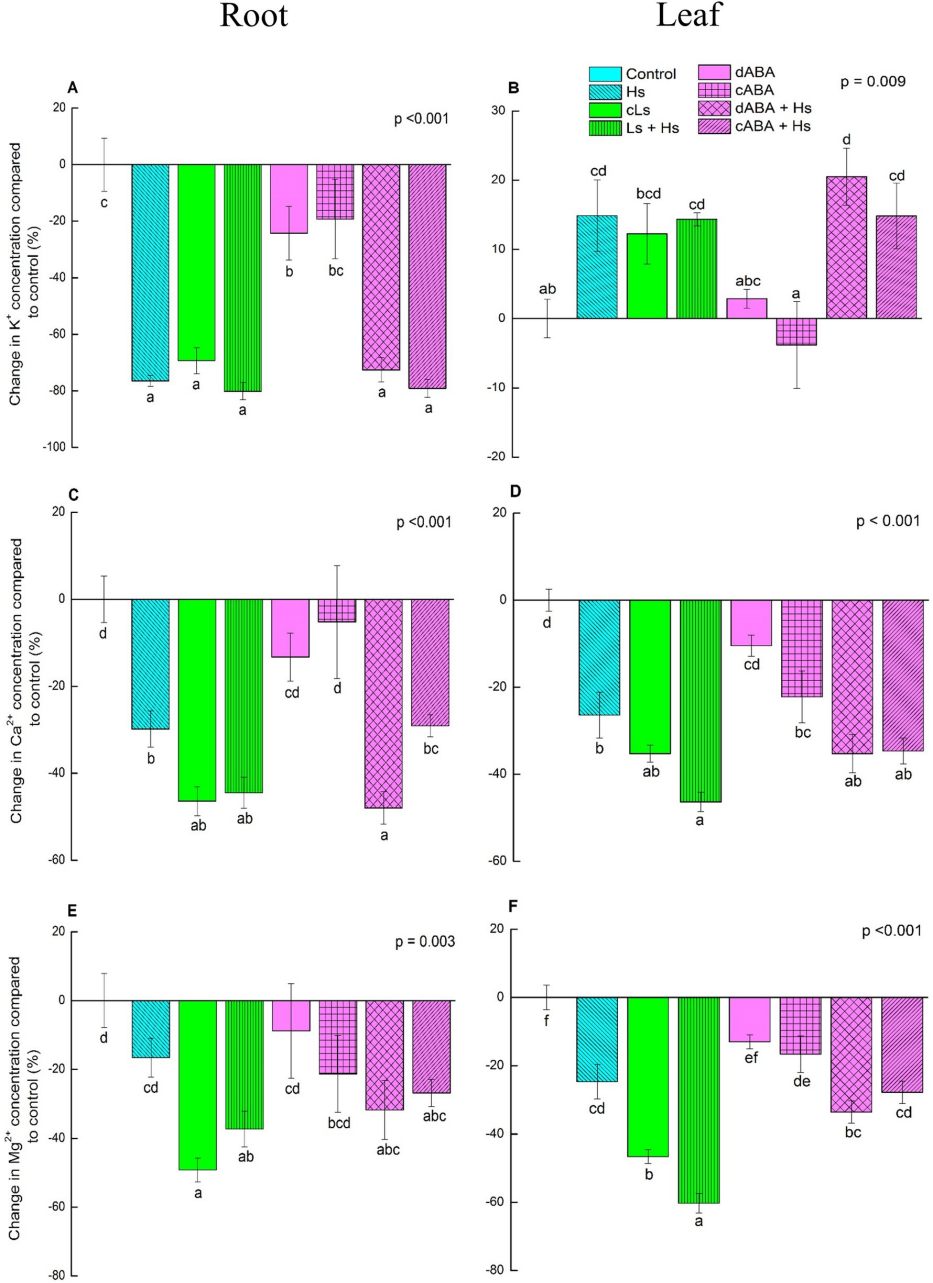
Changes in the concentration of most abundant cations (K^+^, Ca^2+^ and Mg^2+^) in root and leaf tissues in response to salinity and ABA. Graph A, C and E represent the changes in K^+^, Ca^2+^ and Mg^2+^ concentrations respectively compared to control in roots. Graph B, D and F represent the changes in K^+^, Ca^2+^ and Mg^2+^ concentrations respectively compared to control in leaves. In case of respective ion, mean value of controls was subtracted from each treatment value and then % change compared to control condition was calculated. Bar represents means ± SE (n = 5 or 10). For statistical analysis, all treatment values got after subtracting mean value of control were subjected for one-way ANOVA. Different letters obtained from Fisher’s test indicate significant differences among treatments at p <0.05.

To inspect the influence of salinity and ABA on the major cations K^+^, Ca^2+^ and Mg^2+^ in greater details, we analyzed the relative changes in comparison to controls conditions (Fig 4). In roots, salt treatments resulted in almost 80% K^+^ loss, regardless of low or high salt stress (Fig 4A), while the foliar K^+^ level even showed a significant increase (Fig 4B). Interestingly, ABA treatments also resulted K^+^ reduction in roots, although relatively moderate (Fig 4A), whereas no increase was observed in leaves (Fig 4B). Combined treatment of ABA und salt resulted in a K^+^ level comparable to the salt only treatment.

The concentrations of Ca^2+^ were greatly decreased in response to both high salt and low salt treatments (almost -50%) in roots (Fig 4C), while leaf Ca^2+^ levels were more reduced under high salt (up to -46%) than under low salt stress (-35%) (Fig 4D). ABA treatment in the absence of salt stress caused also decline in Ca^2+^ concentrations in leaves, but not in roots (Fig 4C and 4D).

The stress treatments tended to decrease the Mg^2+^ levels in roots compared to controls but the effects were only significant for low salt stress (cLs) and low salt stress followed by high salt stress (Ls+Hs) (Fig 4E). In leaves, the negative effect of high salt on the Mg^2+^ level was more pronounced than in roots and other treatments. The concentrations of Mg^2+^ in leaves were also decreased significantly in ABA treatments (Fig 4F).

### Accumulation, but not radial distribution, of cations in root cells declines in response to salinity

**Fig 5:**
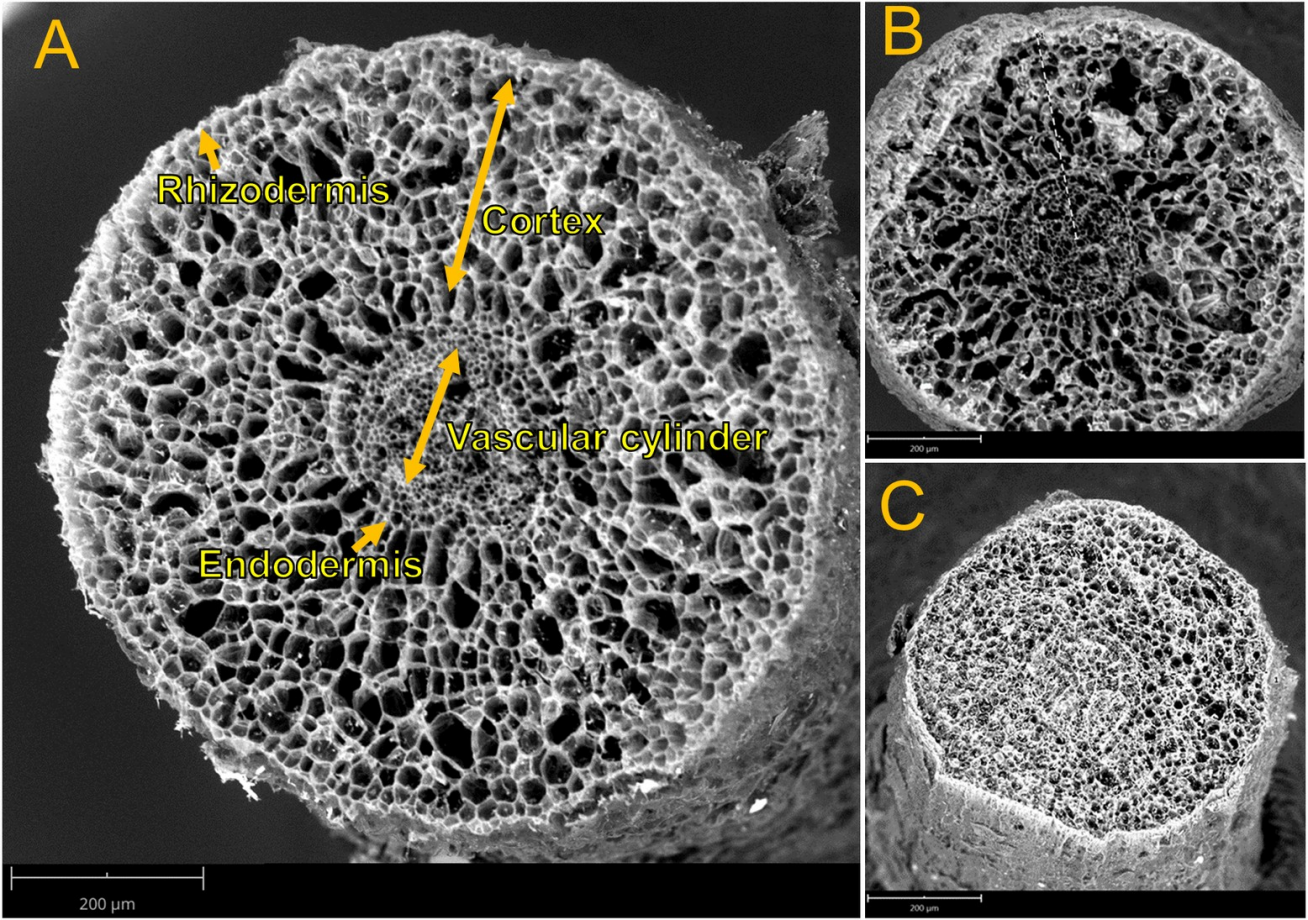
SEM imaging of *P*. × *canescens* root. Cross section (A) showing different cell layers in root. Sections of root from 100 mM NaCl treated plant (B) as well as control plant (C) are presented. Bars = 200 μm.

Restricting the radial movement of ions across the root greatly reduces the amounts loaded into xylem for delivery to upper tissues. Therefore, distribution of ions radially from outer cortex to the endodermis (denominated cortex) and the inner vascular cells of roots were analyzed by SEM-EDX (Fig 5). Root samples from salt treated plants as well as control plant were compared (Fig 5). Elemental analysis in cortex and vascular cells of fine roots revealed that relative levels of Na^+^, K^+^, Ca^2+^ and Mg^2+^ did not significantly vary between cortex and vasculature under any of the observed treatments (Table 1). In contrast, relative concentrations of Cl^-^ were moderately decreased in vascular cells compared to the cortex in response to salt stress. The relative accumulation of Na^+^ and Cl^-^ in both cells were increased in the presence of high and low salt stress and the increases were greater in high salt stress than in low salt stress (Table 1). In contrast, the relative concentration of K^+^ decreased significantly in both cortex and vascular cells in response to high and low salt exposure but the response was less pronounced for low than for high salt stress (Table 1). Ca^2+^ levels showed similar decreases, regardless of high or mild salt treatments (Table 1). Mg^2+^ levels were unaffected by any salt treatments (Table 1). The distribution of other elements (S, P and Mn) between the cortex and vascular system and their responses to salinity are shown in S6 Table.

**Table 1:** Relative concentration of Na, K, Ca, Mg and Cl in the cortex and the vascular tissues of root of *P*. × *canescens* from EDX analysis.

Tissue	Treatment	Relative element concentration (weight %)				
		Na	K	Ca	Mg	Cl
Cortex	Control	5.59 ± 0.65 a	49.91 ± 4.02 c	8.08 ± 0.33 b	7.59 ± 0.47 abc	3.57 ± 0.41 a
	Hs	31.65 ± 4.29 d	14.91 ± 4.60 a	4.84 ± 0.27 a	6.79 ± 0.43 abc	10.06 ± 0.73 bc
	cLs	20.18 ± 3.93 bc	35.59 ± 4.79 b	5.66 ± 1.15 a	4.96 ± 1.46 a	12.01 ± 1.89 cde
	Ls + Hs	32.26 ± 3.77 d	19.70 ± 3.74 a	4.46 ± 0.57 a	5.44 ± 1.75 ab	12.42 ± 1.45 cde
	cABA + Hs	35.77 ± 4.08 d	16.96 ± 5.38 a	3.98 ± 0.41 a	4.85 ± 1.23 a	15.08 ± 2.32 e
Vascular tissue	Control	5.60 ± 0.39 a	49.52 ± 3.72 c	7.80 ± 0.34 b	8.00 ± 0.40 c	3.45 ± 0.39 a
	Hs	28.38 ± 4.32 cd	13.96 ± 4.48 a	4.94 ± 0.36 a	7.36 ± 0.33 bc	8.94 ± 0.32 b
	cLs	19.98 ± 3.59 b	34.43 ± 4.47 b	4.97 ± 1.25 a	5.35 ± 1.40 abc	9.89 ± 1.44 bc
	Ls + Hs	29.88 ± 4.24 d	18.71 ± 3.52 a	4.46 ± 0.50 a	5.76 ± 1.66 abc	10.50 ± 0.36 bcd
	cABA + Hs	34.04 ± 3.16 d	15.85 ± 4.73 a	3.97 ± 0.25 a	5.78 ± 1.61 abc	13.54 ± 2.33 de
p-value	p(treatment)	<0.001	<0.001	<0.001	0.009	<0.001
	p(tissue)	0.38	0.74	0.61	0.44	0.03
	p(treatment × tissue)	0.99	0.999	0.92	0.999	0.98

Data represent mean ± SE (n = 3 or 4) (four measurements were taken from each plant). Two—way ANOVA was conducted for each element with treatment and tissue as two main factors. Beta regression model was used for ANOVA and homogenous subsets were found using Fisher´s test. Different lowercase letters in the column of specific element for both tissues indicate significant differences among treatments at p <0.05.

## Discussion

### Salinity and ABA decreases gas exchange which eventually exerts negative effects on the growth of plant

As an immediate response to osmotic stress caused by salinity, stomatal aperture decreases in salt stressed plants [1]. Decrease in stomatal opening eventually reduces CO_2_ diffusion from the atmosphere to the site of carboxylation which is an immediate cause for decreased photosynthesis under salt stress [30]. In the present study, significant reduction in stomatal conductance was observed in response to high and low salt exposure which eventually reduced net CO_2_ assimilation for photosynthesis and subsequent water loss via transpiration. Decreased gas exchange was also found in response to ABA with no salt exposure, since ABA promotes stomatal closure [31].

Significant decline in shoot height and stem diameter increment in high salt stressed plants in this study was most likely a result of reduced carbon fixation due to very low photosynthesis. Moderate reduction in photosynthesis found under low salt exposure negatively affected shoot growth as well. But the decrease in gas exchange in response to ABA treatment did not affect stem elongation significantly. However, whole-plant leaf area was negatively affected by both salt and ABA treatments. Decreased leaf size in response to salt and ABA contributed to the reduction of whole-plant leaf area here. Leaf shedding is a water-stress avoidance strategy in plants [32, 33], and is controlled by the interplay of phytohormones, including ABA [33, 34]. Besides, ABA is also involved in other morphological changes for acclimation to low water availability such as decreased shoot growth, leaf size and increased root growth [35–37]. Since the root to shoot ratio increased and loss of leaf biomass was not significant in the present study, the negative effect of salt and ABA on whole-plant biomass was moderate.

### Leaf K^+^ level is maintained under salt stress, whereas leaf Ca^2+^ and Mg^2+^ levels are reduced by the influence of transpiration

Accumulated Na^+^ in root and leaf tissues in response to high and low salt stress eventually increased the total cation concentrations. Overall, leaves contained higher cation concentrations than roots under any stress conditions compared to non-stressed plants. The increased concentration may be necessary to maintain water uptake by decreasing osmotic pressure [38]. The decrease in root K^+^ under salinity is most likely the outcome of competition between K^+^ and Na^+^ for uptake [38–40]. Na^+^ competes with K^+^ for the binding site of high affinity (KUP and HKT) K^+^ channels as well as low affinity non-selective cation channels [41, 42]. Moreover, Na^+^ influx into the cells leads to membrane depolarization resulting in leakage in voltage-gated outward-rectifying channels which leads to K^+^ loss [43]. However, this reduction of K^+^ content in the root has no effect on the radial transport towards the central cylinder as we did not observe any restriction in radial translocation of K^+^ from cortex to vascular cells in the root under salt stress. This suggests that xylem loading of K^+^ was unaffected [44]. The dramatic reduction in K^+^ content in the roots did not negatively affect the K^+^ content in leaves. K^+^ even increased under salt stress conditions in leaves in our study. Maintaining or elevating K^+^ levels in leaves is a known mechanism of halophytic plants [45] and has been reported for wheat, barley [46, 47]as well as in earlier studies for *P*. x *canescens* [19] and *P*. *tomentosa* [21]. Thus, we may speculate that a high leaf K^+^ level is an evolutionary conserved mechanism of plants to acclimate to salt stress.

In contrast, salt exposure caused reduced concentrations of Ca^2+^ and Mg^2+^ in roots as well as in leaves, indicating a clear difference between K^+^ on the one hand and Ca^2+^/Mg^2+^, when it comes to the translocation of these elements from the root to the shoot. ABA treatment in the absence of salt did not alter Ca^2+^and Mg^2+^ levels in roots, but lead to a significant reduction in leaves. This observation implies that ABA did not have any negative effect on influx of Ca^2+^ and Mg^2+^ into roots, but had negative effects on the transport from root to shoot. It is known that Ca^2+^ is relatively immobile within the plant and supply to the young tissues is strongly dependent on the current acquisition from the growth medium via transpiration stream [48–50]. Moreover, both Ca^2+^ and Mg^2+^ contents in the shoots of barley seedlings (*Hordeum vulgare*) were reduced due to a decrease in transpiration rate [51]. Therefore, it is highly likely that ABA induced decrease in transpiration rate was a reason for decreased translocation of Ca^2+^ and Mg^2+^ from root to shoot resulting in reduced leaf content of these two elements.

Moreover, reduction in leaf Ca^2+^ level in ABA treated plants (cABA, -22%) was as strong as in high salt stress (Hs, -26%), although transpiration rate was comparatively higher in ABA treatments. This phenomenon suggests a strong influence of transpiration on the ion transport to the leaves under salinity. Since salt exposure increases ABA levels in the plant [13, 14, 18, 19], participation of ABA in the transpiration-based ion transport reduction might also exist in the salt treatments. Although we cannot exclude an influence of our treatments on the transport systems for these cations, our findings indicate that lower transpiration rate induced by salinity plays role in the suppression of Ca^2+^ and Mg^2+^ transport to the leaves.

In summary, reduced transpiration under salinity did not decrease the accumulation of Na^+^ or K^+^ in leaves, suggesting a rather transpiration independent translocation to the leaves. Ca^2+^ and Mg^2+^ levels in leaves under salt stress were at least partially dependent on reduced transpiration rate. Therefore, the present study suggests that the influence of transpiration on foliar accumulation of nutrients in *P*. × *canescens* under salinity is rather modest.

## Supporting information

S1 Fig Flow diagram showing the experimental design. *P*. × *canescens* plants (n = 94) were obtained by micropropagation and then those were grown in hydroponic culture. After five weeks of growth, treatment application was initiated and it was done in two phases: pretreatment and stress. For pretreatment, all plants were divided to grow under three different conditions:–(a) control, (b) 25 mM NaCl and (c) 50 μM ABA. After three weeks of pretreatment, groups were further divided into total 8 treatment groups (each having 9 to10 plants) to stress with NaCl (25 mM or 100 mM) and ABA in different combinations. Plants were treated for three weeks during stress phase. Abbreviated form of each treatment name is given below the respective treatment.
(TIF)

S1 Table Leaf size, leaf biomass loss and root to shoot ratio of *P*. × *canescens* plants under different treatments. Values represent means ± SE (n = 9 or 10; except 7 or 8 in case of leaf size). One-way ANOVA was conducted for each parameter. Normal distribution of data was tested by plotting residuals. Different letters obtained from Fisher’s test indicate significant differences among treatments at p <0.05.
(DOCX)

S2 Table Concentration of different elements measured in the root of *P*. × *canescens* plant grown under different treatments. Values represent means ± SE (n = 5 or 10). One-way ANOVA was conducted in case of each element. Normal distribution of data was tested by plotting residuals and log transformation (in case of K and Ca) or square root transformation (in case of Na) was used to meet these criteria. Homogeneous subsets were found after Fisher’s test. Different lowercase letters in a column indicate significant differences at p <0.05.
(DOCX)

S3 Table Ratios of Na/K, Na/Ca, Na/Mg, Na/Mn, Na/Fe, Na/P and Na/S in the root tissues of *P*. × *canescens* plant grown under different treatments. The ratio was calculated from concentration (mg g^-1^ dry mass) values of the elements. Values represent means ± SE (n = 5 or 10). One-way ANOVA was conducted in every case. Normal distribution of data was tested by plotting residuals and log transformation was used in each case, except Na/S where square root transformation was used to meet these criteria. Homogeneous subsets were found after Fisher’s test. Different lowercase letters in a column indicate significant differences at p <0.05.
(DOCX)

S4 Table Concentration of different elements measured in the leaf of *P*. × *canescens* plant grown under different treatments. Values represent means ± SE (n = 5 or 10). One-way ANOVA was conducted in case of each element. Normal distribution of data was tested by plotting residuals and log transformation was used for certain cases (Na and Fe) to meet these criteria. Homogeneous subsets were found after Fisher’s test. Different lowercase letters in a column indicate significant differences at p <0.05.
(DOCX)

S5 Table Ratios of Na/K, Na/Ca, Na/Mg, Na/Mn, Na/Fe, Na/P and Na/S of in the leaf tissue of *P*. × *canescens* plant grown under different treatments. The ratio was calculated from concentration (mg g^-1^ dry mass) values of the elements. Values represent means ± SE (n = 5 or 10). One-way ANOVA was conducted in every case. Normal distribution of data was tested by plotting residuals and log transformation was used in each case to meet these criteria. Homogeneous subsets were found after Fisher’s test. Different lowercase letters in a column indicate significant differences at p <0.05.
(DOCX)

S6 Table Relative concentration of Mn, S and P in the cortex and the vascular tissues of root of *P*. × *canescens* according to EDX analysis. Data represent mean ± SE (n = 3 or 4) (four measurements were taken from each plant). Two—way ANOVA was conducted for each element with treatment and tissue as two main factors. Beta regression model was used for ANOVA and homogenous subsets were found with Fisher´s test. Different lowercase letters in the column of specific element for both tissues indicate significant differences among treatments at p <0.05.
(DOCX)
