# Supplementary figures and images for: The influence of transpiration on foliar accumulation of salt and nutrients under salinity in poplar (Populus × canescens)
Source: PLoS One. 2021 Jun 24;16(6):e0253228. doi: 10.1371/journal.pone.0253228 (PMC8224899; doi:10.1371/journal.pone.0253228)

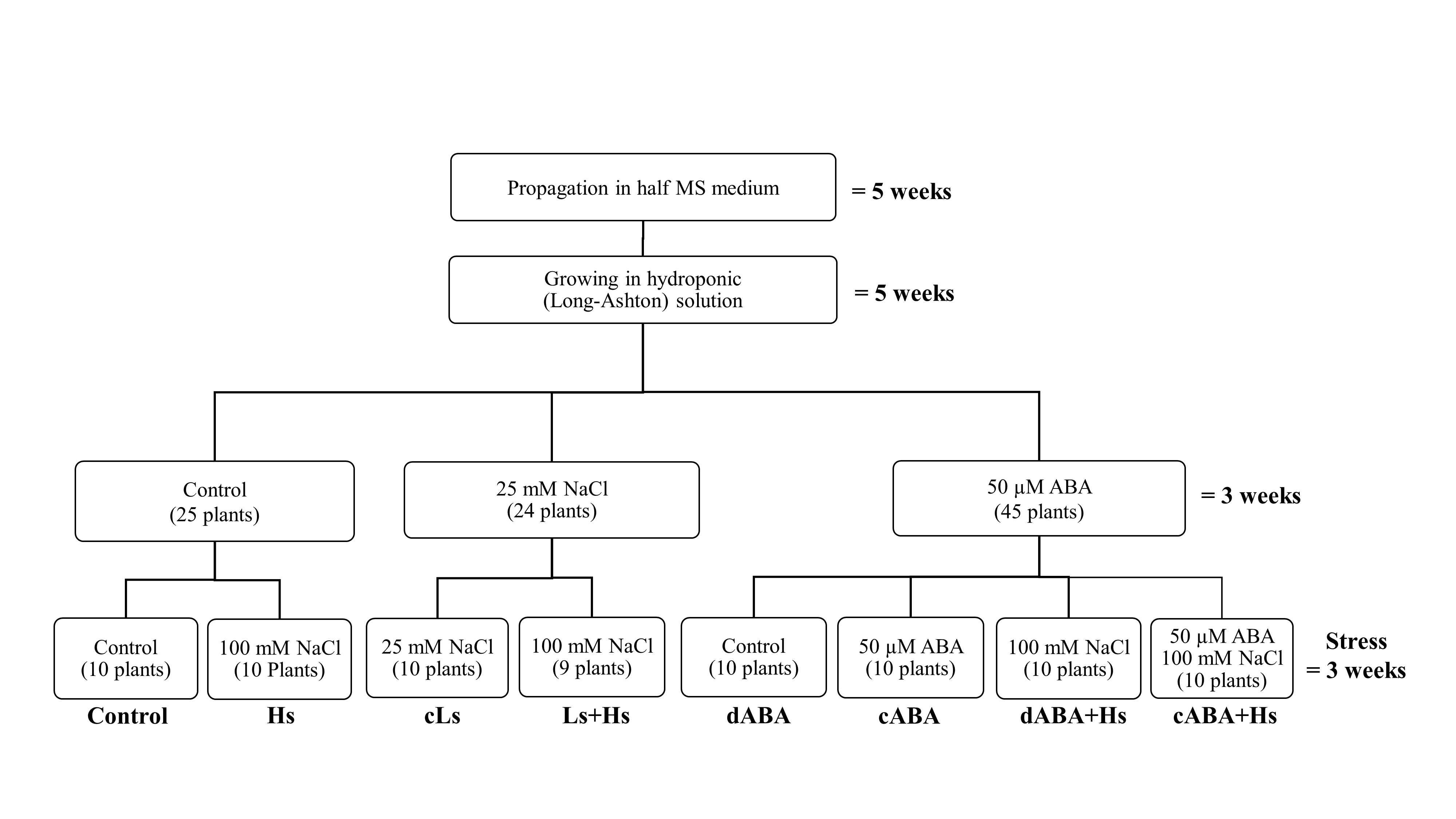

Supplement: S1 Fig — P. × canescens plants (n = 94) were obtained by micropropagation and then those were grown in hydroponic culture. After five weeks of growth, treatment application was initiated and it was done in two phases: pretreatment and stress. For pretreatment, all plants were divided to grow under three different conditions:–(a) control, (b) 25 mM NaCl and (c) 50 μM ABA. After three weeks of pretreatment, groups were further divided into total 8 treatment groups (each having 9 to10 plants) to stress with NaCl (25 mM or 100 mM) and ABA in different combinations. Plants were treated for three weeks during stress phase. Abbreviated form of each treatment name is given below the respective treatment. (TIF) [file pone.0253228.s001.tif]
