# Supplementary material for: The influence of transpiration on foliar accumulation of salt and nutrients under salinity in poplar (Populus × canescens)
Source: PLoS One. 2021 Jun 24;16(6):e0253228. doi: 10.1371/journal.pone.0253228 (PMC8224899; doi:10.1371/journal.pone.0253228)
Supplement: S1 Table — Values represent means ± SE (n = 9 or 10; except 7 or 8 in case of leaf size). One-way ANOVA was conducted for each parameter. Normal distribution of data was tested by plotting residuals. Different letters obtained from Fisher’s test indicate significant differences among treatments at p <0.05. (DOCX) [file pone.0253228.s002.docx]

| **Treatment** | **Leaf size**  **(cm^2^ leaf^-1^)** | **Leaf biomass loss (g plant^-1^)** | **Root/shoot** |
| --- | --- | --- | --- |
| Control | 124.12 ± 7.03 c | 0.42 ± 0.12 a | 0.28 ± 0.01 a |
| Hs | 103.11 ± 3.12 ab | 0.50 ± 0.27 a | 0.31 ± 0.01 a |
| cLs | 107.92 ± 1.79 ab | 0.12 ± 0.05 a | 0.29 ± 0.01 a |
| Ls+Hs | 100.31 ± 5.42 a | 0.31 ± 0.23 a | 0.28 ± 0.02 a |
| dABA | 118.42 ± 7.71 bc | 0.26 ± 0.11 a | 0.30 ± 0.02 a |
| cABA | 101.90 ± 4.87 a | 0.45 ± 0.16 a | 0.32 ± 0.01 a |
| dABA+Hs | 93.62 ± 6.63 a | 0.42 ± 0.19 a | 0.33 ± 0.02 a |
| cABA+Hs | 97.01 ± 5.65 a | 0.79 ± 0.26 a | 0.34 ± 0.03 a |
