# Supplementary material for: The influence of transpiration on foliar accumulation of salt and nutrients under salinity in poplar (Populus × canescens)
Source: PLoS One. 2021 Jun 24;16(6):e0253228. doi: 10.1371/journal.pone.0253228 (PMC8224899; doi:10.1371/journal.pone.0253228)
Supplement: S2 Table — Values represent means ± SE (n = 5 or 10). One-way ANOVA was conducted in case of each element. Normal distribution of data was tested by plotting residuals and log transformation (in case of K and Ca) or square root transformation (in case of Na) was used to meet these criteria. Homogeneous subsets were found after Fisher’s test. Different lowercase letters in a column indicate significant differences at p <0.05. (DOCX) [file pone.0253228.s003.docx]

| **Treatment** | **Concentration of elements in the root tissue** | | | |
| --- | --- | --- | --- | --- |
|  | **Na**  **(mg g^-1^ dry mass)** | **K**  **(mg g^-1^ dry mass)** | **Ca**  **(mg g^-1^ dry mass)** | **Mg**  **(mg g^-1^ dry mass)** |
| Control | 0.32 ± 0.12 a | 4.11 ± 0.38 c | 4.32 ± 0.22 d | 1.11 ± 0.07 c |
| Hs | 15.47 ± 0.67 c | 0.79 ± 0.06 a | 2.47 ± 0.11 b | 0.76 ± 0.04 a |
| cLs | 8.52 ± 0.46 b | 1.81 ± 0.30 b | 3.26 ± 0.13 c | 0.80 ± 0.05 ab |
| Ls+Hs | 16.85 ± 1.43 c | 0.88 ± 0.11 a | 2.65 ± 0.23 b | 0.77 ± 0.05 ab |
| dABA | 0.43 ± 0.06 a | 3.33 ± 0.19 c | 4.07 ± 0.16 d | 1.08 ± 0.07 c |
| cABA | 0.35 ± 0.09 a | 3.44 ± 0.48 c | 4.26 ± 0.35 d | 0.91 ± 0.08 b |
| dABA+Hs | 16.21 ± 1.62 c | 1.00 ± 0.08 a | 2.04 ± 0.09 a | 0.68 ± 0.03 a |
| cABA+Hs | 14.29 ± 0.82 c | 0.76 ± 0.11 a | 2.73 ± 0.13 b | 0.72 ± 0.02 a |
| **Treatment** | **Fe**  **(mg g^-1^ dry mass)** | **Mn**  **(mg g^-1^ dry mass)** | **P**  **(mg g^-1^ dry mass)** | **S**  **(mg g^-1^ dry mass)** |
| Control | 0.60 ± 0.08 a | 0.20 ± 0.05 a | 3.53 ± 0.40 ab | 3.23 ± 0.28 a |
| Hs | 0.58 ± 0.03 a | 0.17 ± 0.01 a | 3.86 ± 0.17 ab | 4.31 ± 0.24 bc |
| cLs | 0.56 ± 0.02 a | 0.14 ± 0.02 a | 3.90 ± 0.25 abc | 4.10 ± 0.30 abc |
| Ls+Hs | 0.73 ± 0.17 a | 0.21 ± 0.03 a | 4.66 ± 0.28 c | 5.38 ± 0.42 d |
| dABA | 0.69 ± 0.05 a | 0.24 ± 0.03 a | 3.15 ± 0.11 a | 3.36 ± 0.20 a |
| cABA | 0.68 ± 0.07 a | 0.16 ± 0.04 a | 4.14 ± 0.28 bc | 3.76 ± 0.39 ab |
| dABA+Hs | 0.52 ± 0.03 a | 0.24 ± 0.04 a | 4.24 ± 0.36 bc | 4.91 ± 0.57 cd |
| cABA+Hs | 0.74 ± 0.04 a | 0.26 ± 0.02 a | 4.20 ± 0.37 bc | 4.82 ± 0.24 cd |
