# Supplementary material for: The influence of transpiration on foliar accumulation of salt and nutrients under salinity in poplar (Populus × canescens)
Source: PLoS One. 2021 Jun 24;16(6):e0253228. doi: 10.1371/journal.pone.0253228 (PMC8224899; doi:10.1371/journal.pone.0253228)
Supplement: S3 Table — The ratio was calculated from concentration (mg g-1 dry mass) values of the elements. Values represent means ± SE (n = 5 or 10). One-way ANOVA was conducted in every case. Normal distribution of data was tested by plotting residuals and log transformation was used in each case, except Na/S where square root transformation was used to meet these criteria. Homogeneous subsets were found after Fisher’s test. Different lowercase letters in a column indicate significant differences at p <0.05. (DOCX) [file pone.0253228.s004.docx]

| **Treatment** | **Ratios of elements in the root tissue** | | | |
| --- | --- | --- | --- | --- |
|  | **Na/K** | **Na/Ca** | **Na/Mg** | **Na/Mn** |
| Control | 0.08 ± 0.03 a | 0.08 ± 0.03 a | 0.32 ± 0.14 a | 1.91 ± 0.68 a |
| Hs | 20.80 ± 2.01 d | 6.40 ± 0.43 c | 20.90 ± 1.46 c | 92.98 ± 5.96 b |
| cLs | 5.17 ± 0.73 c | 2.61 ± 0.07 b | 10.70 ± 0.57 b | 70.77 ± 15.74 b |
| Ls+Hs | 19.53 ± 1.72 d | 6.67 ± 0.97 c | 22.09 ± 1.74 c | 83.32 ± 11.60 b |
| dABA | 0.13 ± 0.01 b | 0.11 ± 0.02 a | 0.39 ± 0.05 a | 1.86 ± 0.31 a |
| cABA | 0.11 ± 0.04 ab | 0.09 ± 0.02 a | 0.41 ± 0.13 a | 3.13 ± 1.41 a |
| dABA+Hs | 16.35 ± 1.46 d | 7.96 ± 0.75 c | 24.03 ± 2.60 c | 72.27 ± 10.36 b |
| cABA+Hs | 19.96 ± 1.96 d | 5.32 ± 0.45 c | 19.84 ± 1.32 c | 55.85 ± 2.07 b |
| **Treatment** | **Na/Fe** | **Na/P** | **Na/S** |  |
| Control | 0.55 ± 0.17 a | 0.10 ± 0.03 a | 0.10 ± 0.04 a |  |
| Hs | 26.97 ± 1.27 c | 4.09 ± 0.26 d | 3.62 ± 0.09 d |  |
| cLs | 15.41 ± 1.37 b | 2.20 ± 0.09 c | 2.10 ± 0.13 b |  |
| Ls+Hs | 28.93 ± 6.67 bc | 3.59 ± 0.12 d | 3.13 ± 0.11 c |  |
| dABA | 0.64 ± 0.11 a | 0.13 ± 0.02 b | 0.13 ± 0.01 a |  |
| cABA | 0.53 ± 0.13 a | 0.08 ± 0.02 a | 0.10 ± 0.03 a |  |
| dABA+Hs | 31.42 ± 2.42 c | 3.88 ± 0.34 d | 3.36 ± 0.21 cd |  |
| cABA+Hs | 19.58 ± 1.72 bc | 3.48 ± 0.28 d | 2.98 ± 0.16 c |  |
