# Supplementary material for: The influence of transpiration on foliar accumulation of salt and nutrients under salinity in poplar (Populus × canescens)
Source: PLoS One. 2021 Jun 24;16(6):e0253228. doi: 10.1371/journal.pone.0253228 (PMC8224899; doi:10.1371/journal.pone.0253228)
Supplement: S4 Table — Values represent means ± SE (n = 5 or 10). One-way ANOVA was conducted in case of each element. Normal distribution of data was tested by plotting residuals and log transformation was used for certain cases (Na and Fe) to meet these criteria. Homogeneous subsets were found after Fisher’s test. Different lowercase letters in a column indicate significant differences at p <0.05. (DOCX) [file pone.0253228.s005.docx]

| **Treatment** | **Concentration of elements in the leaf tissue** | | | |
| --- | --- | --- | --- | --- |
|  | **Na**  **(mg g^-1^ dry mass)** | **K**  **(mg g^-1^ dry mass)** | **Ca**  **(mg g^-1^ dry mass)** | **Mg**  **(mg g^-1^ dry mass)** |
| Control | 0.03 ± 0.00 a | 10.82 ± 0.49 bcd | 13.90 ± 0.49 d | 3.08 ± 0.09 d |
| Hs | 8.10 ± 1.05 c | 9.43 ± 0.41 a | 7.76 ± 0.48 b | 1.75 ± 0.09 b |
| cLs | 2.80 ± 0.18 b | 11.33 ± 0.53 cd | 8.39 ± 0.27 b | 1.54 ± 0.03 b |
| Ls+Hs | 11.49 ± 0.94 d | 9.69 ± 0.41 ab | 5.85 ± 0.36 a | 0.96 ± 0.07 a |
| dABA | 0.04 ± 0.00 a | 12.21 ± 0.26 d | 13.67 ± 0.36 d | 2.95 ± 0.05 d |
| cABA | 0.04 ± 0.00 a | 9.93 ± 0.50 abc | 10.37 ± 0.75 c | 2.46 ± 0.06 c |
| dABA+Hs | 6.98 ± 1.12 c | 9.89 ± 0.56 abc | 6.86 ± 0.61 ab | 1.56 ± 0.12 b |
| cABA+Hs | 7.35 ± 0.98 c | 8.66 ± 0.65 a | 6.34 ± 0.47 a | 1.55 ± 0.10 b |
| **Treatment** | **Fe**  **(mg g^-1^ dry mass)** | **Mn**  **(mg g^-1^ dry mass)** | **P**  **(mg g^-1^ dry mass)** | **S**  **(mg g^-1^ dry mass)** |
| Control | 0.11 ± 0.02 a | 0.07 ± 0.01 bc | 6.21 ± 0.76 b | 3.31 ± 0.20 c |
| Hs | 0.12 ± 0.04 a | 0.07 ± 0.00 bc | 3.69 ± 0.30 a | 2.09 ± 0.10 a |
| cLs | 0.08 ± 0.01 a | 0.09 ± 0.01 d | 3.62 ± 0.26 a | 2.60 ± 0.21 b |
| Ls+Hs | 0.07 ± 0.01 a | 0.08 ± 0.01 cd | 3.20 ± 0.38 a | 2.07 ± 0.15 a |
| dABA | 0.13 ± 0.04 a | 0.08 ± 0.01 cd | 5.51 ± 0.45 b | 4.05 ± 0.20 d |
| cABA | 0.08 ± 0.01 a | 0.06 ± 0.01 ab | 3.92 ± 0.53 a | 3.61 ± 0.07 cd |
| dABA+Hs | 0.07 ± 0.01 a | 0.05 ± 0.01 a | 3.66 ± 0.51 a | 2.47 ± 0.03 ab |
| cABA+Hs | 0.08 ± 0.01 a | 0.05 ± 0.00 a | 2.66 ± 0.26 a | 2.11 ± 0.28 ab |
