# Supplementary material for: The influence of transpiration on foliar accumulation of salt and nutrients under salinity in poplar (Populus × canescens)
Source: PLoS One. 2021 Jun 24;16(6):e0253228. doi: 10.1371/journal.pone.0253228 (PMC8224899; doi:10.1371/journal.pone.0253228)
Supplement: S5 Table — The ratio was calculated from concentration (mg g-1 dry mass) values of the elements. Values represent means ± SE (n = 5 or 10). One-way ANOVA was conducted in every case. Normal distribution of data was tested by plotting residuals and log transformation was used in each case to meet these criteria. Homogeneous subsets were found after Fisher’s test. Different lowercase letters in a column indicate significant differences at p <0.05. (DOCX) [file pone.0253228.s006.docx]

| **Treatment** | **Ratios of elements in the leaf tissue** | | |
| --- | --- | --- | --- |
|  | **Na/K** | **Na/Ca** | **Na/Mg** |
| Control | 2.59 × 10^-3^ ± 1.26 × 10^-4^ a | 2.01 × 10^-3^ ± 8.57 × 10^-5^ a | 0.01 ± 4.09 × 10^-4^ a |
| Hs | 0.88 ± 0.12 c | 1.06 ± 0.13 d | 4.65 ± 0.57 d |
| cLs | 0.25 ± 0.03 b | 0.34 ± 0.02 c | 1.83 ± 0.13 c |
| Ls+Hs | 1.19 ± 0.09 d | 2.01 ± 0.23 e | 12.35 ± 1.67 e |
| dABA | 2.92 × 10^-3^ ± 2.24 × 10^-4^ a | 2.6 × 10^-3^ ± 1.82 × 10^-4^ ab | 0.01 ± 9.1 × 10^-4^ ab |
| cABA | 3.65 × 10^-3^ ± 1.85 × 10^-4^ a | 3.53 × 10^-3^ ± 2.36 × 10^-4^ b | 0.01 ± 1.16 × 10^-3^ b |
| dABA+Hs | 0.73 ± 0.13 c | 1.05 ± 0.17 d | 4.66 ± 0.86 d |
| cABA+Hs | 0.87 ± 0.12 cd | 1.17 ± 0.14 d | 4.74 ± 0.50 d |
| **Treatment** | **Na/Mn** | **Na/Fe** |  |
| Control | 0.39 ± 0.03 a | 0.27 ± 0.03 a |  |
| Hs | 122.66 ± 17.35 d | 86.65 ± 12.25 d |  |
| cLs | 31.11 ± 3.19 c | 33.65 ± 2.71 c |  |
| Ls+Hs | 143.69 ± 15.42 d | 159.51 ± 15.39 e |  |
| dABA | 0.44 ± 0.03 ab | 0.35 ± 0.09 ab |  |
| cABA | 0.66 ± 0.05 b | 0.49 ± 0.07 b |  |
| dABA+Hs | 139.33 ± 26.12 d | 105.53 ± 19.78 de |  |
| cABA+Hs | 158.09 ± 22.90 d | 104.56 ± 22.88 de |  |
| **Treatment** | **Na/P** | **Na/S** |  |
| Control | 4.82 × 10^-3^ ± 7.45× 10^-4^ a | 0.01 ± 3.31 × 10^-4^ a |  |
| Hs | 2.41 ± 0.45 d | 3.87 ± 0.42 d |  |
| cLs | 0.78 ± 0.06 c | 1.11 ± 0.12 b |  |
| Ls+Hs | 3.93 ± 0.76 e | 5.58 ± 0.39 e |  |
| dABA | 0.01 ± 8.79 × 10^-4^ ab | 0.01 ± 9.66× 10^-4^ a |  |
| cABA | 0.01 ± 1.7 × 10^-3^ b | 0.01 ± 8.97× 10^-4^ a |  |
| dABA+Hs | 2.17 ± 0.57 d | 2.83 ± 0.46 c |  |
| cABA+Hs | 2.89 ± 0.47 de | 3.53 ± 0.29 cd |  |
