# Supplementary material for: The influence of transpiration on foliar accumulation of salt and nutrients under salinity in poplar (Populus × canescens)
Source: PLoS One. 2021 Jun 24;16(6):e0253228. doi: 10.1371/journal.pone.0253228 (PMC8224899; doi:10.1371/journal.pone.0253228)
Supplement: S6 Table — Data represent mean ± SE (n = 3 or 4) (four measurements were taken from each plant). Two—way ANOVA was conducted for each element with treatment and tissue as two main factors. Beta regression model was used for ANOVA and homogenous subsets were found with Fisher´s test. Different lowercase letters in the column of specific element for both tissues indicate significant differences among treatments at p <0.05. (DOCX) [file pone.0253228.s007.docx]

| **Tissue** | **Treatment** | **Relative element concentration (weight %)** | | |
| --- | --- | --- | --- | --- |
|  |  | **Mn** | **S** | **P** |
| Cortex | Control | 3.39 ± 0.23 c | 7.74 ± 0.26 a | 8.10 ± 1.68 ab |
|  | Hs | 2.91 ± 0.04 abc | 7.65 ± 1.04 a | 10.03 ± 0.87 bcd |
|  | cLs | 2.50 ± 0.36 a | 6.58 ± 1.81 a | 6.32 ± 1.32 a |
|  | Ls+Hs | 2.91 ± 0.28 abc | 6.98 ± 1.12 a | 10.77 ± 2.27 bcd |
|  | cABA+Hs | 2.66 ± 0.06 ab | 6.59 ± 2.04 a | 8.80 ± 0.43 abc |
| Vascular  tissue | Control | 3.32 ± 0.10 c | 7.69 ± 0.18 a | 9.29 ± 2.03 abc |
|  | Hs | 3.37 ± 0.22 c | 8.30 ± 0.71 a | 13.19 ± 1.47 de |
|  | cLs | 2.68 ± 0.29 ab | 6.22 ± 1.16 a | 10.70 ± 0.98 bcd |
|  | Ls+Hs | 3.15 ± 0.33 bc | 7.02 ± 1.38 a | 15.58 ± 1.47 e |
|  | cABA+Hs | 3.00 ± 0.17 bc | 6.92 ± 1.71 a | 11.62 ± 0.66 cde |
| p-value | p(treatment) | <0.001 | 0.29 | <0.001 |
|  | p(tissue) | 0.045 | 0.73 | <0.001 |
|  | p(treatment × tissue) | 0.74 | 0.99 | 0.57 |
